# Pulling Mobile Assisted Language Learning (MALL) into the Mainstream: MALL in Broad Practice

**DOI:** 10.1371/journal.pone.0128762

**Published:** 2015-05-26

**Authors:** Qun Wu

**Affiliations:** School of Foreign Languages, Jiujiang University, Jiujiang, Jiangxi Province, China; University of Akron, UNITED STATES

## Abstract

The researcher designed a smartphone app to help college students to learn English (L2) vocabulary. The app contained 3,402 English words that were compiled into an alphabetic wordlist with each word displayed on three features; namely: spelling, pronunciation and Chinese definitions. To test the effectiveness of the app, an experimental group (with app) was compared with a control group (without app) and knowledge of words was tested before and after the research. The study revealed that the students using the program significantly outperformed those in the control group in vocabulary acquisition. This paper introduced a research design method and set up a pedagogical paradigm which can be followed as a way to practice MALL.

## Introduction

[1] concluded that a vocabulary size of 8,000–9,000 word families is necessary for reading and an amount of 5,000–7,000 word families for oral address is required in English. Whereas, the Chinese college students plateau at 3,934 English words [2] by the end of their second academic year. The 3,934 words were calculated with a standardized survey method established by the Chinese Ministry of Education from a glossary pool of 6,674 words that included simple words like “I”, “in”, “one”, etc. Applying the same survey technique, [3] discovered that the productive vocabulary amount for sophomores was 2,000 words in a top Chinese university. Most Chinese college students receive 11 years of English education (3 years in elementary school, 6 years in junior and senior high schools and 2 years in college), in other words, they are taught English for a total of 1,672 class hours (based on an estimate of 38 class weeks per year, 4 English class hours each week). Researchers [4] also found that Chinese college students spent more than half of their own study time on learning English. Compared to [1]’s requirements, the daunting gaps in vocabulary size and the tremendous amount of time being spent on learning the language perplex many Chinese English learners to look for shortcuts if there is any, and spur teachers on to search for useful vocabulary acquisition techniques.

As the ineffectiveness of traditional vocabulary learning techniques being well documented [5–7], English as a second language (ESL) researchers exert to modern technology to devise new methods. Some researchers [8–13] turned to mobile phones and proposed “pushing” and “access” theories. By sending short text messages at spaced intervals, ESL students are pushed to learn English vocabulary. Since a mobile phone is always in one’s pocket, its convenient accessibility is superior to that of textbooks and computers, etc. The effectiveness of learning English vocabulary or idioms via mobile phones was also empirically investigated by [10] in Japan, [14] in Taiwan, [15] in Turkey and [16] in Iran; these researches showed that mobile technology enhanced learning.

However, all these empirical experiments were designed to utilize very small texts with few words or idioms sent via short message service (SMS), e.g., there were merely 14 words in one week [14]. The obvious deficiency in SMS is the size of the message. Even where mobile carriers allow subscribers to send large files, few learners will have enough patience to scroll down a small screen and keypad to study huge content without a format for any length of time. Another deficiency in SMS method is in delivering and receiving messages. [10] admitted that only 10% of participants read messages at the time of reception. MALL should be at “anytime, anywhere” [17]. The third deficiency is that traditionally with mobile technology, learners have no learning choice. They wait to receive their lessons; they have no choice of content, and no way to access further material when they have mastered what they have received to date. The usefulness of any design with these three shortcomings should be questioned. [18] regards these SMS studies and other MALL researches as class trials, deems the value of these designs as marginal in practical implementation and believes there is technological foundation to pull MALL in from the fringes to the mainstream of foreign language learning.

This researcher believes that it is the functional shortcomings of mobile phones that limited these SMS researches in employing mobile phones for foreign language learning to be conducted with specifically tailored small content in artificial environments. Given the advances in mobile technology, smartphones and appropriate apps can overcome the three deficiencies.

A smartphone is built with an operating system. Recent smartphone models add the functionality of portable media players, high-resolution touchscreens and web browsers that display standard web pages. The selection of smartphones to conduct this study was based on four criteria: First, the technology; smartphones are designed with fast operating systems, big display screens, large internal storage, and touchscreen technology with a zoom function. Touchscreen with zoom allows users to react to what is displayed and to control how it is displayed by zooming (i.e. expanding or shrinking text size). Second is the popularity of smartphones; the majority of students possess smartphones (personal survey: all 143 students attending the researcher’s courses carry smartphones). China Mobile, the largest wireless carrier with the most subscribers in the world, goes to Chinese universities to offer free smartphones for a low price subscription plan. A student can obtain a smartphone on a subscription plan of 30 Yuan/month (1 USD = 6.10 Yuan). Third, custom made applications such as the one this researcher designed can be installed into smartphones to solve the small content problem existing in these SMS studies. Regular mobile phones, without operating systems, do not support third party applications. Finally, with extensive searching this researcher found few experiments conducted with smartphones to teach/learn vocabulary in the field of language learning. Of the few researches involving smartphones for language learning, they were positioned at either interacting instructors with learners in a Q/A type simple design through MSN and SMS [19], or tutoring kids to look for a word at smartphone’s online dictionary [20]. I believe it is the scarcity of available curriculum content suitable for smartphones that hampers them being fully exploited and being as useful as PCs in language learning. If proper teaching/learning materials are designed, this problem could be rectified. I designed an application with broad practical usage to exploit MALL with smartphones in daily life, without any modification and intervention.

Basic4Android (B4A), very similar to Visual Basic, is a simple programming language designed to be easy to learn and use. B4A is the simplest and most powerful rapid application development tool available for the Android platform [21]. Users can create graphical user interface applications simply by using components supplied by B4A itself without any coding.

Wordlist method, defined as “by working through a list of L2 words together with their L1 translations and memorizing the word-gloss pairs” ([22], p. 40), is the prevailing technique adopted by 90% of Chinese college students [23] to learn English vocabulary. It has theoretical merits. In a conversation about the ten best ways for ESL students to learn vocabulary, two distinguished ESL educators suggested wordlist and word cards, respectively [24]. [25] and [26] concluded that there are a minimum number of encounters with a word needed to recognize its morphological form. [27] detected the neural mechanism of repetition to advance better memorization and stated “repeated study improves memory”. Wordlist method can generate many repetitions or encounters with words in a short time.

The importance of passing College English Test-Band 4 (CET4) is not emphasized enough. Many universities will not issue a diploma until the student passes CET4 in China. A lot of students failed to pass the test because they did not master these CET4 words [4]. To help students to prepare for this test, an astounding number of vocabulary books have been printed. A “CET4 vocabulary book” key word search in an e-commerce website (www.taobao.com) on March 22, 2015 returned 790,312 results. In searching for a new way to help learners to learn, and for this project, I referred to fifteen CET4 vocabulary books and four English-Chinese dictionaries. From these books and the book *College English Curriculum Requirements*, I collected 3,402 words to form a database which was then incorporated into the smartphone vocabulary acquisition application, also designed by the researcher, called Word Learning-CET4.

To solve the three deficiencies in the aforementioned SMS studies so as to render MALL to practical usage, to make learning English vocabulary more conveniently and help students to pass the CET4, coupled with the four listed criteria, I selected smartphone instead of old mobile phone as the tool, chose the simplest programming language B4A to design the app, adopted the widely employed wordlist technique to help learning, further, incorporated the tool, the app and the technique as a seamless learning project.

This study was conducted by using empirical research method to investigate the effectiveness of the learning project in helping ESL college students learn English vocabulary without intervention. The present study aimed to:

1. Explore the effectiveness of using smartphones as a tool for learning English vocabulary in a natural environment. It was based on the assumption that the participants with Word Learning-CET4 installed in their smartphones would have more encounters with these words due to its accessibility,
2. Introduce a pedagogical example of how to build a smartphone learning project for learning vocabulary.

## Methods

The Ethics Committee of Jiujiang University deemed the research was part of classroom teaching and the experiment could be conducted without its permission. Because of that, a written consent was not required to be presented; however, all participants orally agreed to take part in the research. “All participants orally agreed” was documented in an application to the committee and it authorized the procedure.

### Participant selection

English is a required course for all Chinese college freshman and sophomore students and is selectively taught according to students’ competence in most Chinese universities. Upon arrival to Jiujiang University the students are settled into three different placements (Good, Average and Poor) based on their English scores in the National College Entrance Examination (CEE).

The participant selection was to find two separate intact classes taught by one instructor, with the group of students who possessed Android smartphones in one class displayed no significant difference in knowledge of vocabulary recognition to the corps of students who also held Android smartphones in another class. The chosen two intact classes were tagged as a pair. For the selection, classes with similar number of students who possessed Android smartphones were screened from multiple sophomore “Good” classes. Subsequently, English vocabulary recognition pretest was conducted and three pairs of classes were obtained. Unlike applying for college admission in most countries, a Chinese high school graduate is practically assigned to an university based on his/her CEE score; because of that, all freshmen have statistically identical CEE score in a mid-ranked college as Jiujiang University. The researcher assumed the three pairs of classes had no significant difference in IQ and learning ability.

All participants were being taught English language in the 11^th^ year. There were 47 male and 54 female students in the three randomly selected experimental classes, and 46 males and 52 females in the three control classes. They were sophomores and their age ranged between twenty to twenty-two.

### Origin of vocabulary pool

The Chinese Ministry of Education requires Chinese college students to acquire a vocabulary of 4,538 words by the time of graduation. These words are listed as basic requirements in the book of *College English Curriculum Requirements*, administered by the ministry. To make word lists more compact, many CET-4 vocabulary books were compiled by eliminating simple words like “I”, “in”, and “to”, etc. In a similar design this researcher reduced the 4,538 words to 3,402 words to form the database of this project.

### Word Learning-CET4 application software design

**Fig 1:**
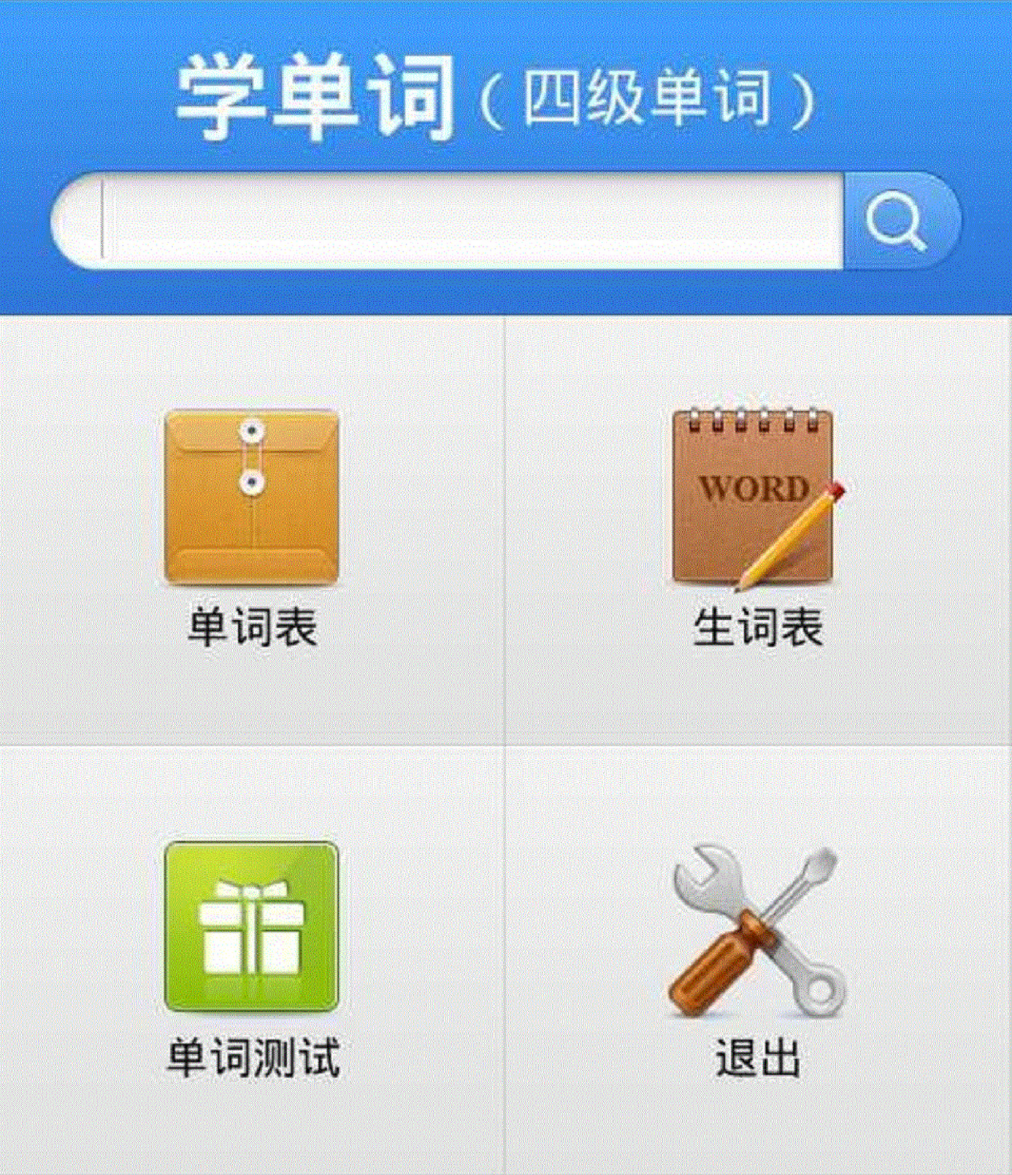
Screenshot image of the app, Word Learning-CET4, designed by the researcher.

In preparation, the 3,402 words were compiled into an applicable computer database with three features; namely: spelling, pronunciation and Chinese definitions. With these focal features, the researcher designed Word Learning-CET4, a B4A application with touchscreen commands (Fig 1. To download the app, please visit the researcher’s blog, http://perry20008.blog.sohu.com).

The reasons for selection of B4A to develop Word Learning-CET4 were:

1. B4A is very simple, easy to learn and use, and very popular in designing apps for Android.
2. Android is the most widely used smartphone operating system. As in July 2012, Android’s market share rose to 90% in China [28].
3. Apps designed with other programming languages for other smartphone operating systems (e.g. Apple iOS) may not be executable on smartphones with Android platform.

### Functions of Word Learning-CET4 application

The Word Learning-CET4 software has the following six commands:

1. Search command: To search for a word from its 3,402 words database.
2. Glossary command: To scroll through the alphabetically listed 3,402 word database.
3. Unknown Words command: A touch screen function that isolates words that are unknown to a user from those that are known. There are three separate folders aptly named Unknown Words One, Two, and Three. Users can load and clear these files at any time. A user can select, as an example, 1,000 words from the glossary and put them in Unknown Words One for repetitive study. From those words the user may select, again as an example, 500 of the more difficult words for Unknown Words Two, and subsequently a reduced number of words from this group of study words that are proving particularly difficult to fill the Unknown Words Three folder. This function allows the user to increase the frequency of learning the more difficult words by eliminating the redundancy of seeing the words already known, thus improving efficiency. Unknown Words folders are automatically built and saved once words are touchscreen selected or de-selected.
4. Sample Test command: To create a sample test. Users can randomly select a number of words (10–200), create a sample test and examine how much they have learned.
5. Touchscreen Selection: Users can finger-touch any part of the definition of a word to select it. It will be de-selected if it is touched again. The display color changes to inform users whether a word is selected or de-selected.
6. Exit command: To exit the application.

### Research design

The selected three pairs of intact classes were taught by three colleagues. Of each pair, one class was randomly chosen to be the experimental class; another class was assigned as the control class. The three experimental classes had 101 students who possessed Android smartphones and they were labeled as the experimental group; the control group consisted of 98 students who held Android smartphones in the three control classes. Word Learning-CET4 was only installed into smartphones in the experimental group and access to it was protected by a password key. The researcher controlled the password and the app could not be reproduced from an unlocked copy.

The experiment was partially conducted with one pair of classes in the Spring semester of 2012–2013 academic year. Thereafter, because the university liked the value of the app and advised to implement it, the researcher easily obtained two more desired pairs of intact classes and compared them in the Spring semester of 2013–2014 academic year. At the beginning, the researcher installed Word Learning-CET4 to their smartphones of the participants in the experimental group and taught them how to use it. The process of installation and teaching lasted less than 30 minutes because it was designed with simple interfaces and the participants were familiar with smartphones. During the entire experiment, the researcher and instructors did not do any intervention, it was up to these participants to decide whether to use it or not. All students in the three pairs of intact classes received printed copies of the 3,402 words in a wordlist to study. The university did not change textbook and the three instructors taught the three pairs of classes with the same curricular textbook, *New Horizon College English*: *Reading and Writing*, *book 4*, *2nd* edition.

### Pretest and posttest

By Sample Test function in Word Learning-CET4, random selections of words were performed twice to extract 100 words each time. These selected words were used to perform the pretest and posttest in an effort to evaluate the vocabulary level of recipients in the research. Examinees were asked to write down the common Chinese meaning during the tests; one point was awarded if a correct Chinese meaning or interpretation was noted.

The pretests were conducted in the first class of semester.

The posttests were conducted in the last class of semester.

## Results

### Estimated time on task

All participants in the experimental group learned vocabulary with Word Learning-CET4 (Table 1). Another finding was that 95 of them acknowledged that they occasionally spent idle time, such as waiting and commuting, on learning.

**Table 1:** Average learning hours participants spent on with Word Learning-CET4.

Hours	<0.5 hour/day	0.5–1.0 hr/d	>1.0 hr/d
**No. of Participants**	53	37	11

### Comparison of the experimental group and control group before and after treatment (pretest and posttest)

Besides the treatment, time was also a variable since it took a semester to conduct the experiment. Participants might increase their vocabulary because of such a long period of time to learn. To determine which variable contributed to the increase, a 2 x 2 two way ANOVA statistics was performed. This analysis was found that there was statistically significant difference for the Time x Group interaction test (F (1, 398) = 8.44, *p* = .004).

**Fig 2:**
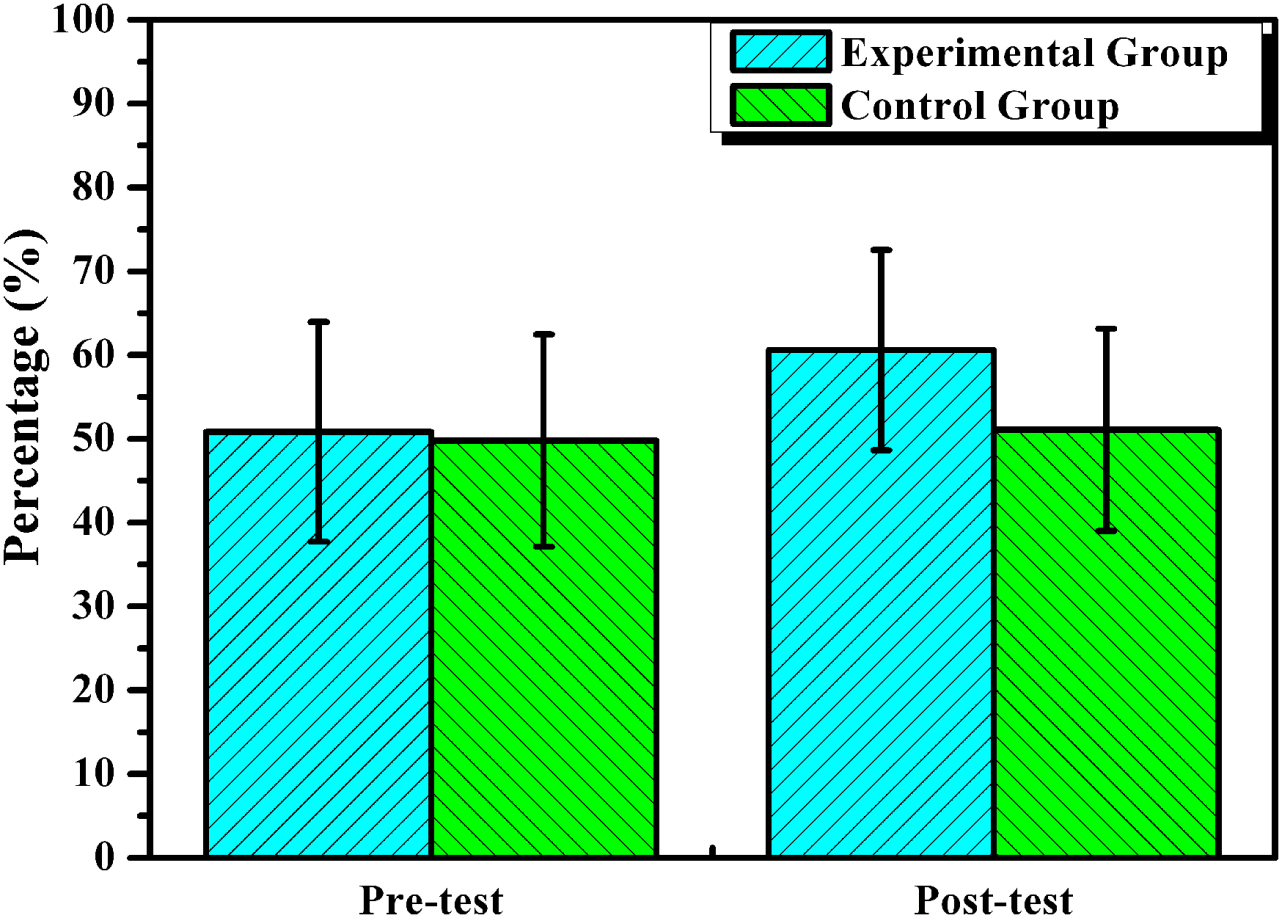
Comparisons of vocabulary means (in percentages) of the experimental group and control group in the pre-test and post-test.

After one semester treatment, the mean (60.61) (Table 2) of the experimental group was significantly greater than the mean (51.08) of the control group in the posttest (*p* = 0.000) while they displayed no significant difference in the pretest (*p* = 0.568) (Fig 2). Subsequently, to analyze the effect of the app, score differences (Posttest—Pretest) in groups were calculated and compared. The mean increase of 9.77 (60.61–50.84) in the experimental group was significantly greater than the mean increase of 1.28 (51.08–49.80) in the control group (*p* = .001).

**Table 2:** Descriptive group statistics, the Mean ± SD.

	Experimental Group (n = 101)	Control Group (n = 98)
**Pretest**	50.84 ± 13.11	49.80 ± 12.68
**Posttest**	60.61 ± 11.96	51.08 ± 12.08

Since the treatment word pool of each test comprised 100 words, the mean score in Table 2 and Fig 2 could be interpreted in percentage terms. That is, the participants in the experimental group recognized 8.49% (9.77–1.28 = 8.49) more of these 3,402 words than the participants in the control group due to the contribution of Word Learning-CET4.

## Discussion

The result of a 8.49% augmentation that the participants in the experimental group remembered over the participants in the control group in the posttest of a test size of 100 words was significant. An increase of 8.49% is meaningful within the context of passing the crucial CET4 exam because college students have a small vocabulary and many fail near the gate. All respondents recognized an average of 2,400 words and 32% respondents scored between 50–59 points (60 to pass) at a CET4 exam in one university [29]. Considering that each pair of participants were taught by one instructor and there was no significant difference before the treatment, the usefulness of adopting smartphones with Word Learning-CET4 for ESL learners to learn English vocabulary was recorded. This study provides additional support to [30]’s conclusion that “mobile technology can enhance learners’ second language acquisition”.

The higher score may be attributed to three reasons. First, providing the participants in the experimental group with direct and explicit information about words in a simple wordlist design may have enabled them to improve their vocabulary acquisition by reducing memorization burden. In other words, the participants could focus on the three key features of the words; i.e., spelling, pronunciation and Chinese definitions; they would not be disturbed by other contents, such as synonyms, antonyms and usage examples which are often included in vocabulary books. Word Learning-CET4 is in essence a compiled list of 3,402 words that electronically mimics a paper word list, but is more accessible and convenient than paper word lists. It is also more appealing to modern day language learners.

The second reason that the participants in the experimental group were more successful may be the Word Learning-CET4 functions. Functions like Unknown Words and Sample Test. Unknown Words helps users to concentrate on these words they find difficult to learn, it saves time and improves learning efficiency; Sample Test assists learners to evaluate their learning progress.

The third reason, and probably the most important, was the length of the time the research took. It was conducted over one full academic semester. In such a long period of time, the participants in the experimental group were likely to be motivated and facilitated with frequent engagement in vocabulary learning [31] and spent a longer accumulated time on learning these 3,402 words because of the convenient access they had to the material. The participants may have subconsciously spent more time on learning by "taking advantage of 'dead time', for example, when commuting" [32]. The finding (section 3.1) that most of the participants in the experimental group sometimes spent their idle time on learning corroborates this argument. Longer accumulated study time results in more repetitions and repetitions lead to better recollection [27], [33]. Traditionally, more repetitions require more effort. With Word Learning-CET4, however, some of this effort is alleviated through convenience, access, and ease of use. As such, "dead time" becomes active time. On the other hand, the participants in the control group had to have their textbooks, word cards, self recorded mp3 files, or word lists on hand to take advantage of this "dead time". As a result, they would often find themselves in situations where they had time to study but could not because they did not have access to the material. That material was lying idle at the end of their bed. As the experiment went on, this researcher and the instructors witnessed that the participants in the experimental group spent their idle time on learning the words via their smartphones.

Compared to studies of [10], [14–16], mentioned earlier, on sending SMS texts to learn English words or idioms, this researcher believes her design solved the three deficiencies existing in their researches. Firstly, the technological constraints limited their material to small content, i.e. 14 words in one week [14]. The broad practical application of learning like this is quite dubious. Word Learning-CET4, on the other hand contains 3,402 words in a 359k application file with neat formatting and simplicity. Secondly, since Wording Learning-CET4 is designed to be installed into smartphones directly, it can be utilized at “anytime, anywhere”; there is no access or delivery problem. On the other hand, learners in their studies could do nothing but to wait passively for SMS messages at set times, on set days, of which punctual delivery and reception were not well known. E.g., in [10], only 10% participants read SMS on time. Lastly, Word Learning-CET4 has the options to selectively choose which words you need to concentrate on with Unknown Words and to evaluate your performance with Sample Test. Word Learning-CET4 meets the standards of “apps based” set by [34] and “anytime, anywhere” by [17] in MALL activities. That the participants spent time (Table 1) on learning with their smartphones certifies its value.

Unlike contents that were artificially tailored into short blocks to be readable in above mentioned researches, the Layout of Word Learning-CET4, with the words are listed alphabetically in a wordlist formation, is comparable to a vocabulary book. Word Learning-CET4 is natural. The design of the experiment that the participants in the experimental group received no additional treatment except with downloaded Word Learning-CET4 was also natural. Consequently, the researcher claims that smartphones are effective supplementary tools in addition to textbooks for participants to learn English vocabulary in a natural environment.

Although electronic Chinese-English dictionaries/translators are incorporated in apps of some smartphone models, or, users can download or directly access online dictionaries or buy a commercial smartphone app if their smartphones do not come with a dictionary application, this researcher’s design still has significance. A dictionary is a tool that is designed for users to search for unknown words that they encounter in daily life. It is hard, if not impossible for users to pick up to actually learn these words and put them into daily use because the database is so large that revisiting these words is difficult, sporadic, and laborious. Word Learning-CET4 is designed to get words into peoples' daily vocabulary. It is designed for word acquisition, not word recognition. It has six simple commands designed to make word acquisition seamless, effortless, and efficient. It is based on the specific vocabulary students are required to study for their university careers.

## Pedagogical implication

Apart from being an example for further MALL activities, the pedagogical implication of this study may give educators, textbook writers or publishing houses certain ideas in which, in the future, they may include files or applications downloadable to smartphones along with their materials. This researcher believes that it will become increasingly important for designing educational materials in technical fields, where learners must understand a range of specific terms and their underlying meanings to successfully operate in the workplace of their chosen field, to work safely and professionally, and for those with ambition, to advance. A smartphone is always handy. Readily available, in case of uncertainty, performers are more likely to pull out their smartphones to search for solution than risk dangerous attempt if practical apps have been installed into. Depending on the content of the downloadable file, and specifically for teachers, if a downloadable file is not accompanied with a textbook and designing a complete application is a difficult challenge, teachers can simply make a. doc or PDF file for students to download into their smartphones.

This researcher considers that the design of Word Learning-CET4 was a simple task. Her knowledge of computer sciences was limited, but with a limited amount of assistance it was quite manageable. Its simple B4A interfaces were designed by the researcher, an English teacher with a B4A manual (the researcher downloaded it from Internet). The only time consuming part was the repetitive work of collecting, organizing and compiling the 3,402 words. This mundane work is easy to be hired out. In conclusion, for the pedagogical portion of this study, this researcher believes that most teachers can make their own mobile teaching/learning materials in a similar or perhaps better design. The intention of this portion of the article is to introduce a direction in which educators should be setting their minds, and to set up a pedagogical example which can be followed.

## Limitations of the study

The improvement should be done is to upgrade the Word Learning-CET4 application. The researcher discovered the absence of a function to communicate with users; another missing function is update, for correcting errors or supplying new content. Both are requisites to run live class. Future research design should allow participants to study with either a list of words on paper or a smartphone for a set amount of time. This research presents a neonatal idea to invite further scrutiny.

## Conclusion

The first purpose of this study was to find a new way for Chinese students to improve their ability to retain English vocabulary. The design was to make learning vocabulary more convenient and user friendly. To do this, Word Learning-CET4 was created. Word Learning-CET4 is a simple word learning program using B4A, based on the curriculum for the students involved in the study, and readily accessible using a smartphone. The goal was to show that access to the proper material, convenience of use, and the integration of advances in modern technology with an old school method could significantly improve vocabulary acquisition. Using Word Learning-CET4 the participants in the experimental group remembered 8.49% more words than the participants in the control group. This is a significant result and this researcher believes there are three reasons for it. The first two are the convenience and accessibility; the third is the technological functions incorporated into the program that make learning, testing and reviewing easy.

The second purpose of this study was to pave the way for educators of second language acquisition to better results. Learning from past researchers, repetition, accessibility and convenience are all important aspects of learning a language. The smartphone and B4A programming language provide an easy solution for all of those obstacles. This researcher hopes that in reading this paper other educators would embrace this new technology while doing their service to simply make learning English as a second language easier.
